# The HOPS complex subunit VPS39 controls ciliogenesis through autophagy

**DOI:** 10.1093/hmg/ddaa029

**Published:** 2020-02-20

**Authors:** Daniela Iaconis, Claudia Crina, Simona Brillante, Alessia Indrieri, Manuela Morleo, Brunella Franco

**Affiliations:** 1 Telethon Institute of Genetics and Medicine, 80078 Pozzuoli, Italy; 2 Medical Genetics, Department of Translational Medical Sciences, University of Naples Federico II, 80131 Naples, Italy; 3 Institute for Genetic and Biomedical Research, National Research Council, 35 20122 Milan, Italy

## Abstract

Primary cilia are microtubule-based organelles that assemble and protrude from the surface of most mammalian cells during quiescence. The biomedical relevance of cilia is indicated by disorders ascribed to cilia dysfunction, known as ciliopathies, that display distinctive features including renal cystic disease. In this report, we demonstrate that vacuolar protein sorting 39 (VPS39), a component of the homotypic fusion and vacuole protein sorting (HOPS) complex, acts as a negative regulator of ciliogenesis in human renal cells, by controlling the localization of the intraflagellar transport 20 protein at the base of cilia through autophagy. Moreover, we show that VPS39 controls ciliogenesis through autophagy also *in vivo* in renal tubules of medaka fish. These observations suggest a direct involvement of the HOPS complex in the regulation of autophagy-mediated ciliogenesis and eventually in target selection. Interestingly, we show that the impact of autophagy modulation on ciliogenesis is cell-type dependent and strictly related to environmental stimuli. This report adds a further tile to the cilia-autophagy connection and suggests that VPS39 could represent a new biological target for the recovery of the cilia-related phenotypes observed in the kidneys of patients affected by ciliopathies.

## Introduction

Primary cilia protrude from plasma membranes of almost all mammalian cells and represent highly dynamic organelles that assemble when cells exit cell cycle (1, 2). These organelles are composed by a microtubule-based structure, the axoneme, which forms by nucleation from the basal body that originates from the mother centriole of the centrosome (3, 4). Primary cilia lack protein synthesis and rely on the intraflagellar transport (IFT) and the trafficking machinery to dynamically deliver or to remove ciliary components to ensure cilia biogenesis, axoneme elongation and cilia maintenance (5). Cilia play critical roles in various biological processes such as motility, sensory perception and signaling (6) by sensing the extracellular environment and detecting mechanical forces (e.g. fluid flow in kidney tubules), signaling molecules (e.g. hedgehog ligands and growth factors), chemical compounds (e.g. olfactory molecules) or physical cues (e.g. light on the retina) (7). Ciliogenesis is thus highly influenced by extracellular stimuli and serum starvation is widely used to induce ciliogenesis in cultured confluent cells (8).

The biomedical relevance of cilia is demonstrated by a specific class of disorders, known as ciliopathies, due to mutations in genes encoding proteins localized at cilia and/or necessary for cilia formation, maintenance and function (9). Ciliopathies include rare and more common conditions [e.g. Oro-Facial-Digital type I (OFDI) syndrome and autosomal dominant polycystic kidney disease (ADPKD), respectively] with distinctive features such as cystic disease affecting kidneys, liver, biliary tract and pancreas, and developmental anomalies (9).

The molecular mechanisms that trigger cilia formation and axonemal extension are not fully understood. In silico network-based analysis dissecting the cilia/centrosome complex interactome showed that many ciliary proteins are involved in protein synthesis, protein folding and degradation (10). Recent studies showed that primary cilia control macroautophagy, (hereafter autophagy) and that, conversely, autophagy is one of the main players in the regulation of ciliogenesis (11–17). Autophagy is a self-degradative process necessary for balancing sources of energy during development and in response to nutrient stress. During autophagy induction, a number of autophagy-related proteins (ATGs) orchestrate the formation of autophagosomes, membranous organelles instrumental for this process (18, 19). Membrane targeting of the LC3 protein is essential for autophagosome formation and LC3 detection is widely used to monitor autophagy-related processes (20). The final steps of autophagy include fusion of autophagosomes with lysosomes to generate autophagolysosomes in which content degradation results in recycling of nutrients back into the cytoplasm. This process degrades the cytosolic material such as organelles and aggregates, whereas selective autophagy involves recognition and removal of specific targets, and is achieved through autophagy receptors, such as p62 and NIDP52, that recognize specific cargoes with whom are then degraded inside lysosomes (reviewed in 21).

The link between autophagy and primary cilia is not well defined. It has been shown that autophagy promotes ciliogenesis through selective degradation of the centriolar satellites pool of the OFD1 protein (11). This protein is codified by the *CXORF5* transcript (22), which was subsequently named *OFD1* when found to be mutated in the OFD type I syndrome (23). OFD1 localizes at centrosome/basal bodies (24–26) and displays a critical role in cilia formation in all tissues analyzed to date (27–31). Conversely, Pampliega *et al*. (32) demonstrated that basal autophagy acts as a negative regulator of ciliogenesis by degrading IFT20, a protein essential for cilia formation and assembly (33). However, data published to date report contrasting results concerning the role of autophagy in ciliogenesis and it has been hypothesized that specific environmental stimuli could finely regulate the interplay between autophagy and ciliogenesis in a cell context dependent manner (see (34) for a review).

Different components are involved in the autophagic cascade including the HOPS complex that, in addition to a well-established role in the endocytic pathway, is also involved in autophagolysosomes formation (35, 36). HOPS is a conserved tethering complex made up of six vacuolar protein sorting (VPS) proteins (VPS11, VPS16, VPS18, VPS33A, VPS41 and VPS39) (35, 37). In particular, VPS39, found associated to late endosomes and lysosomes, promotes endosomes/lysosomes clustering and their fusion with autophagosomes (38–42). In addition, VPS39 has been shown to directly bind LC3 to facilitate autophagosomes/lysosomes tethering in *Caenorhabditis elegans* (43).

We recently demonstrated that the OFD1 protein controls the translation rates of specific mRNAs including VPS39 that represents the most abundantly enriched translational target (26). In addition, we showed that VPS39 protein levels increase in mutant kidneys of two different murine models of inherited renal cystic disease (i.e. OFDI and ADPKD) (26). These observations prompted us to investigate the role of VPS39 in cilia dynamics. The *in vitro* and *in vivo* data, here presented, demonstrate that VPS39 acts as a negative regulator of ciliogenesis through autophagy. Moreover, we also show that VPS39 controls IFT20 and OFD1 localization at the base of cilia in human renal cells.

## Results

### Autophagy inhibits cilia elongation in human renal cells

**Figure 1:**
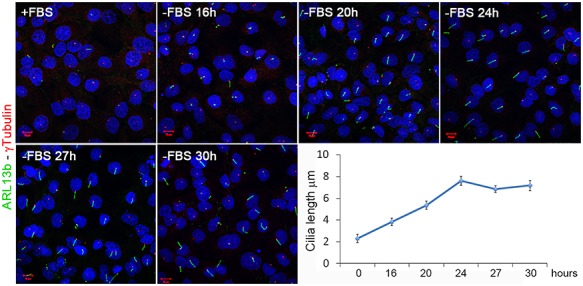
**Cilia dynamics under serum deprivation in HK2 cells.** ARL13b antibody (green) was used to mark primary cilia. Cilia elongated up to 24 h after starvation. After this time the ciliary length remained constant. The basal body was marked with an anti-γ Tubulin antibody (red). The graph shows the quantification of primary cilia length. To evaluate significance One-way Anova test analysis of mean (no equal variance) with post hoc analysis was used (*p-*value < 2.2e–16).

Ciliogenesis is a dynamic process and is strictly associated with cell cycle. Serum starvation can induce cell quiescence (G1/G0) and thus ciliogenesis (44). Cell cycle timing is cell-type dependent and we thus first defined the timing occurring for cilia formation and axoneme elongation in Human Kidney (HK)2 cells. We cultured cells to confluence in serum rich medium and subsequently starved them with serum-deprived medium for 16, 20, 24, 27 and 30 h. We then performed immunofluorescence (IF) with an antibody against ARL13b to mark primary cilia. In this system, cilia were visible 16 h after serum deprivation and elongated up to 24 h (Fig. 1). At later stages (from 24 up to 30 h of serum deprivation) cilia length remained constant (Fig. 1). We thus defined 24 h of starvation as the timepoint needed for HK2 cells to achieve full elongation of cilia. In early starvation (within 24 h) cilia form and elongate, after 24 h the elongation is complete and cilia maintenance takes place.

**Figure 2:**
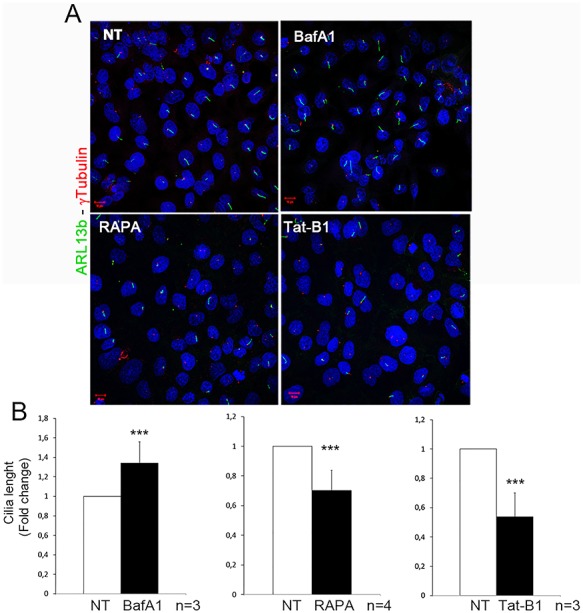
**Autophagy negatively regulates ciliogenesis in HK2 cells.** Serum-deprived cells were treated with autophagy modulators: Bafilomycin A1 (BafA1), Rapamycin (RAPA), Tat-Beclin1 peptide (Tat-B1). In the graphs below the influence of autophagy on cilia length is represented as fold change. In all graphs, white bars correspond to untreated (NT) cells, while black bars represent cells treated with autophagy modulators. Bars = 10 μm. Hoechst (blue) was used to stain nuclei. Data are presented as the mean ± SEM. To evaluate significance t-test (graph in B, ^***^P-value < 0.005) was used. *n* = number of replicates.

To further define the role of autophagy in cilia elongation, we treated HK2 cells with different autophagy modulators: BafilomycinA1 (BafA1), an inhibitor of the autophagosomes-lysosomes fusion, and Rapamycin (RAPA) or the Tat-Beclin1 (Tat-B1) peptide, which induce autophagy. Rapamycin is an inhibitor of the Ser/Thr protein kinase mammalian target of rapamycin, which regulates cell growth and metabolism in response to environmental cues (45) while Tat-B1 acts on Beclin 1 (46), an essential autophagy protein involved in autophagic vesicle nucleation (47). Confluent cells were incubated in serum-deprived medium; drugs were added after 8 h and cells were then incubated for additional 16 h. Our results indicate that treatment with the autophagy inhibitor BafA1 results in longer cilia compared to controls (Fig. 2). Conversely, treatment with both the autophagy inducers RAPA and Tat-B1 results in shorter cilia (Fig. 2). The appropriate controls proved effectiveness of drug treatments and are illustrated in Figure S1A. Contrary to what we observed in HK2, previous reports demonstrated that suppression of autophagy results in impaired ciliogenesis in hTert-RPE1 cells (11, 16, 48, 49) and we confirmed that treatment of hTert-RPE1 cells with BafA1 results in shorter cilia in our experimental conditions (Figure S1B). All together these results demonstrate that autophagy inhibits cilia elongation in HK2 cells, confirming the so far only hypothesized concept that this degradative process acts both as a negative or positive modulator of cilia elongation depending on the cellular context (reviewed in 34).

### VPS39 modulates cilia formation and elongation through autophagy

We hypothesized that VPS39 could influence ciliogenesis, since its overexpression results in autophagy induction, and its silencing inhibits autophagolysosomes formation and consequently blocks autophagy in *Drosophila* larvae and in HeLa cells (38, 50). We first confirmed that VPS39 controls autophagy also in renal cells by evaluating protein levels of LC3II and p62, in *VPS39*-silenced cells and 3XFlag-hVPS39 (ovVPS39) overexpressing cells. Our results indicate that loss of *VPS39* blocks autophagy in HK2 cells, as shown by increased levels of LC3II and p62 protein levels (Figure S2A). Conversely, cells transfected with ovVPS39 and treated with BafA1 showed increased degradation rates of LC3II and p62, suggesting that the autophagic flux is enhanced in VPS39 overexpressing cells compared to controls (Figure S2B).

**Figure 3:**
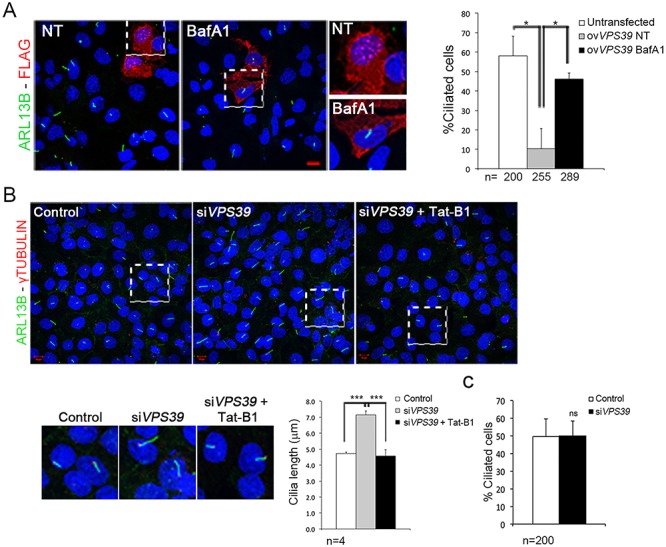
**VPS39 controls cilia formation and elongation in HK2 cells through autophagy.** (**A**) HK2 cells overexpressing 3XFlag-hVPS39 (red) stained with Arl13b antibody (green) do not display primary cilia. Treatment with Bafilomycin (BafA1) recovered cilia. Quantification of ciliated cell is reported in the graph on the right. In total, 15% of cells overexpressing 3XFlag-hVPS39 (ovVPS39) retained the primary cilium (gray bar) compared to the number (60%) of ciliated untransfected cells (white bar). Analysis of untransfected and ovVPS39-treated cells was performed on cells plated on the same slide. Treatment with BafA1 recovered the presence of primary cilia in cells overexpressing 3XFlag-hVPS39 (black bar). (**B**) *VPS39*-silencing resulted in longer cilia (right upper panel) quantified in the graph (gray bar). Cilia length was rescued by treatment with Tat-Beclin1 (Tat-B1, left lower panel and black bar in the graph). The basal body was marked with an anti-γTubulin antibody (red). Bars = 10 μm. Hoechst (blue) was used to stain nuclei. (**C**) Histograms show the quantification of percentage of ciliated cells. No differences were observed between *VPS39*-silenced cells and control. Data are presented as the mean ± SD (graph in A and C) and mean ± SEM (graph in B). Proportion-test (A) and two-way Anova test (B) with *post hoc* analysis were used to evaluate significance ^***^P-value < 0,005; ^*^P-value < 0.05. *n* = number of cells (graph in A and C) and number of replicates (graph in B). ns = not significant.

We then wondered whether modulation of VPS39 protein levels influences ciliogenesis in HK2 cells and examined primary cilia in 24 h-medium starved cells. Our results indicate that VPS39-overexpression had a strong effect on cilia formation, as over 80% of cells did not retain primary cilia (Fig. 3A), and the remaining ciliated cells showed shorter cilia (Figure S3). Consistent with these observations, cilia length increases in *VPS39*-silenced cells compared to controls (Fig. 3B). While the number of ciliated cells did not vary between controls and siVPS39 treated cells (Fig. 3C). These observations suggest that VPS39 is involved in cilia formation and elongation in early phases of starvation (within 24 h) in renal cells. We then asked whether VPS39 could regulate ciliogenesis through autophagy. We thus treated *VPS39*-overexpressing and *VPS39*-silenced cells with BafA1 (Fig. 3A) and the Tat-B1 peptide (Fig. 3B), respectively, and examined cilia by IF. This analysis performed with anti-ARL13b and anti-γtubulin antibodies revealed that autophagy modulators BafA1 and Tat-B1, which exert their function in opposite direction in autophagic processes, rescue the ciliary phenotype in both VPS39-overexpressing and -depleted cells (Fig. 3). Altogether these results demonstrate that VPS39 controls ciliogenesis and cilia elongation through autophagy.

### VPS39 controls localization of IFT20 and OFD1 at the base of cilia

Recent reports demonstrated that autophagy-mediated degradation in the pericentriolar region of specific ciliary targets (e.g. IFT20 and OFD1) modulates ciliogenesis (11, 17, 32). We hypothesized that VPS39 could be involved in the dynamic localization of IFT20 and OFD1 at the base of cilia.

**Figure 4:**
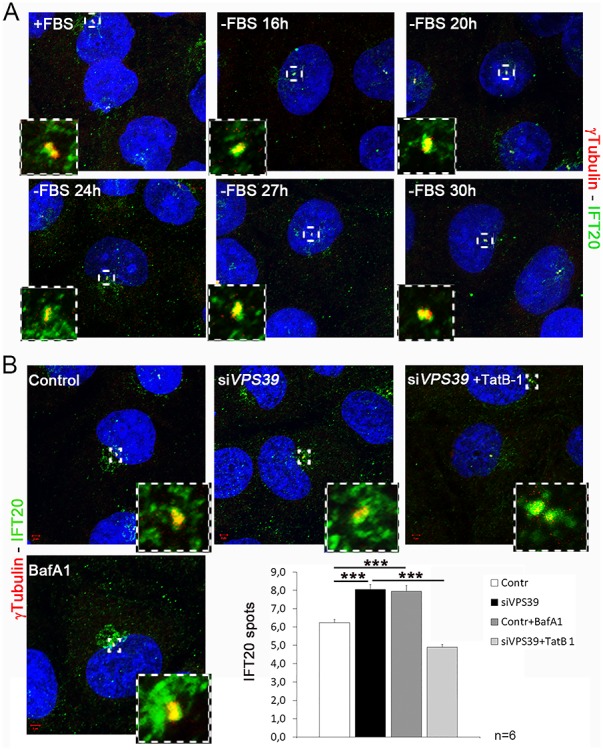
**VPS39 controls localization of the pericentriolar pool of IFT20 trough autophagy.** (**A**) IFT20 centrosomal dynamics in serum deprivation. IFT20 (green) accumulates up to 24 h after serum deprivation and decreases during prolonged starvation around the centrosome, marked with γtubulin (red). (**B**) IFT20 (green) accumulates around the centrosome in *VPS39*-silenced (si*VPS39*) cells (top middle panel and dark gray bar in the graph) and in cells treated with Bafilomycin (BafA1, lower panel and light gray bar in the graph) compared to controls (top first panel and white bar in the graph). The accumulation is rescued in si*VPS39* cells treated with Tat-Beclin1 (TatB-1). Bars = 10 μm. Hoechst (blue) was used to stain nuclei. Data are presented as the mean ± SEM. Two-way Anova test was used to evaluate significance ^***^P-value < 0.005. *n* = number of replicates.

We first analyzed the subcellular localization of IFT20, a positive regulator of ciliogenesis (51) during serum deprivation, and performed IF experiments in wild-type (wt) HK2 cells fixed at 16, 24, 27 and 30 h after serum removal. Our results indicate that IFT20 accumulates after 16 h and up to 24 h of starvation to promote ciliogenesis and decreases during prolonged starvation (after 24 h), to avoid uncontrolled cilia elongation (Fig. 4A). We then asked whether VPS39 could control the levels of IFT20 at the basal body through autophagy to influence ciliogenesis. We first verified by IF in wt cells the accumulation of IFT20 under treatment with BafA1 (Fig. 4B). The increased levels of IFT20 were then confirmed after *VPS39* silencing which, similarly to BafA1 treatment, blocks the autophagic flux. Interestingly, IFT20 levels were rescued by Tat-B1 treatment (Fig. 4B) indicating that VPS39 influences ciliogenesis by controlling IFT20 localization at the base of cilia through autophagy.

**Figure 5:**
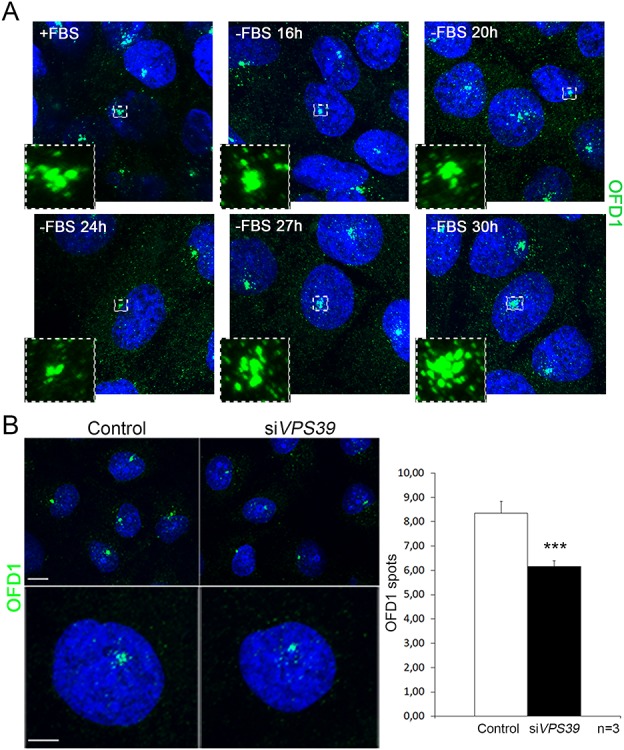
**Loss of VPS39 decreases the satellite pool of OFD1.** (**A**) OFD1 centrosomal dynamics in serum deprivation. OFD1 (green) decreases in the pericentriolar region up to 24 h after serum deprivation and increases during prolonged starvation. (**B**) OFD1 (green) levels decrease around the centrosome in *VPS39*-silenced (si*VPS39*) cells (left panels and black bar in the graph) Bars = 10 μm. Hoechst (blue) was used to stain nuclei. Data are presented as the mean ± SEM. t-test was used to evaluate significance ^***^P-value < 0.005. *n* = number of replicates.

Autophagy-mediated degradation of the pericentriolar pool of OFD1 induces cilia formation in hTert-RPE1 cells and mouse embryonic fibroblasts (MEFs) demonstrating that the pericentriolar pool of OFD1 acts as negative regulator of ciliogenesis (11, 17, 32). We thus evaluated the centriolar satellites distribution of OFD1 in HK2 cells. We first analyzed the dynamic localization of OFD1 during serum deprivation and found that, as expected, the OFD1 pool at centriolar satellites decreases in early starvation to allow cilia formation and accumulates later on to avoid uncontrolled cilia elongation (Fig. 5A). We then quantified the levels of pericentriolar OFD1 in *VPS39-*silenced cells and found it decreased compared to control cells suggesting that VPS39 controls also the pericentriolar levels of OFD1 in HK2 cells (Fig. 5B). All together our results indicate that VPS39 controls the subcellular localization of both positive and negative effectors of ciliogenesis.

### VPS39 influences ciliogenesis in renal tissue through autophagy

**Figure 6:**
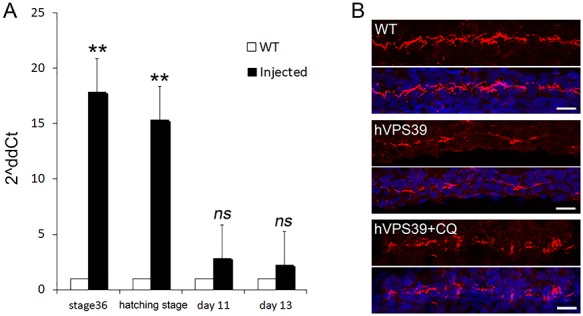
**VPS39 influences ciliogenesis *in vivo*.** (**A**) Expression of human (h) VPS39 mRNA transiently injected in medaka fish embryos (1/2 cell stage). Fish were collected at stage36 and 9-(hatching), 11- and 13-days post-injection and mRNA levels were analyzed by real-time PCR. Expression levels of h*VPS39* were maintained up to day 9 (hatching stage) in injected fish (black bars) compared to controls (white bars). (**B**) IF with acetylated tubulin (red) revealed that h*VPS39* overexpression (middle panels) results in fewer ciliated renal tubule cells compared to controls (upper panels). When fish are treated with CQ an increased number of ciliated cells was detected. DAPI was used to stain nuclei. One tail t-test was used to evaluate significance. Data are presented as the mean ± SEM. ^**^P-value < 0.01.

Cilia dysfunction is commonly observed in models of renal cystic disease and has been described in mutant kidneys of different *Ofd1*-inactivated *in vivo* models (26–28). We asked whether VPS39 could have a role in ciliogenesis *in vivo* and we overexpressed VPS39 in medaka fish (*Oryzias latipes*) by transiently injecting the human *VPS39* (h*VPS39*) mRNA into 1/2 cell stage (st) embryos. Fish were sacrificed at st36 since *VPS39* overexpression is not maintained after hatching stage (Fig. 6A); moreover, at this stage pronephron differentiation is complete (52). We marked primary cilia with an antibody against the acetylated-tubulin previously shown to stain cilia in fish models (53, 54) and IF analysis revealed impaired ciliogenesis in renal tubules of *VPS39*-injected fish compared to controls (Fig. 6B). We finally treated *VPS39*-injected fish with Chloroquine (CQ), a negative regulator of the autophagic flux. CQ-treated fish did not display any gross abnormalities and the treatment did not have evident effect on cilia (data not shown). However, CQ-treated *VPS39*-injected fish showed a partial rescue of the ciliated cells (Fig. 6B) indicating that also *in vivo* VPS39 is able to control ciliogenesis through autophagy.

## Discussion

Recent experimental evidence indicates that ciliogenesis and autophagy are reciprocally regulated (11, 13, 16, 32). However, the identity and defined role of the main players, the timing of action and the conditions in which the intricated cilia-autophagy crosstalk takes place need to be established (34). In this report, we demonstrate for the first time that VPS39 a component of the HOPS complex, with a well characterized function in late endosome-lysosome fusion (38–42), and more recently also implicated in autophagosomes/lysosomes tethering, directly influences ciliogenesis through autophagy both in human renal cells and in *Orizias latipes* renal tubules.

The first and so far, only example of an autophagy component that directly controls ciliogenesis is represented by VPS15 encoding for a regulatory subunit of the class III phosphatidylinositol 3-phosphate lipid kinase VPS34. VPS15 is essential for the formation of UVRAG/Beclin1 and Atg14L/Beclin1 complexes, which are required for membrane trafficking and autophagy, respectively (55, 56). Mutations in the *VPS15* gene result in cilia abnormalities, due to defective formation and/or release of IFT20 positive vesicles from the *cis*-Golgi (57).

Different reports suggest that autophagy positively controls primary cilia formation and elongation in a number of cell lines (RPE, MEFs and human lung cancer cells) (11, 13, 49, 58, 59). In contrast, it has been shown in MEFs that autophagy is not required for cilia formation as Atg5 depleted cells do not display defective cilia that instead developed faster and appeared longer compared to controls upon serum removal (32). Moreover, Struchtrup and colleagues described increased cilia length upon autophagy inhibition in MEFs and reduction of this cilia parameter after autophagy activation in the same cells (48). On the same line, it was demonstrated that exposure to cigarette smoke with consequent enhanced autophagy results in decreased length of epithelial motile cilia (60). Here we show that blocking of the final steps of autophagy promotes cilia elongation in human renal cells, and conversely inhibits ciliogenesis in retinal cells. Altogether these observations suggest that autophagy may have a cell context dependent role in cilia formation and maintenance implying that autophagy players and ciliary cargoes may differ according to the cell-type under study and to the experimental conditions used. Analysis of tissue-specific expression of autophagy players and co-factors could help in understanding why the same biological process has opposed effects in different tissues and could give information about the cell-dependent role of autophagy in mediating protein degradation to regulate ciliogenesis. Moreover, different experimental culturing conditions could influence the reproducibility of experiments and explain the discrepancy of results generated in different laboratories. On the basis of our experience, we propose and recommend that detailed information on the experimental culturing conditions and of the stage of cilia development under study (formation, elongation or maintenance) should always be described in such studies. This would be the only condition to ensure reproducibility of experiments and comparison of results generated in different laboratories.

Cilia impairment and unbalanced autophagic flux have been associated to renal cystic disease (34, 61), one of the commonest inherited disorders for which an effective therapy is still not available. Data generated *in vivo* from different laboratories indicate a role for autophagy in renal physiology. It has been reported that basal autophagy represents a key homeostatic mechanism to maintain proximal tubules/podocyte integrity (62–64). In addition, autophagy, which is upregulated by stress stimuli such as renal ischemia and nephrotoxins, has been described as a surveillance sensor for renal cells (49, 65). Moreover, unbalanced autophagy has been associated to different renal cystic disease mutants. Decreased autophagy has been described in mutant kidneys of an autosomal recessive polycystic kidney disease (ARPKD) murine model (66), and increased autophagy was shown to improve the phenotype in ADPKD zebrafish models (67). On the other hand, increased autophagy has been reported in precystic kidneys in aqua-porin11 null mice (68) and an ARPKD rat model in which pharmacological inhibition of autophagy significantly reduced cysts growth (69). Finally, we now report that impaired ciliogenesis occurs after autophagy induction mediated by VPS39 overexpression in *in vivo* and *in vitro* renal models. Our study now provides a novel player of the cilia-autophagy axis in kidneys, VPS39, whose expression levels are significantly increased in two different murine models of renal cystic disease (26). To the best of our knowledge, this is the first report of a component of the HOPS complex directly involved in ciliogenesis and possibly in renal function.

In conclusion, we now report the characterization of an additional player in the field of autophagy-mediated control of ciliogenesis, the VPS39 protein, which could represent a new biological target for the recovery of the cilia-associated phenotypes observed in kidneys of ciliopathy patients. Moreover, the data here presented open new questions with regards to tissue specificity and selective target degradation in the fine regulation of autophagy-mediated ciliogenesis.

## Material and Methods

### Cell lines

HK2 cells were cultured in DMEM/F-12 (Dulbecco’s modified Eagle’s Medium: Nutrient Mixture F-12; Thermofisher) medium supplemented with 5% of fetal bovine serum (FBS), 1% penicillin and streptomycin (P/S ), 1% ITS (Insulin, Transferrin and Sodium Selenite media supplement, from SIGMA I1884), 1% Glutamine. hTert-RPE-1 (Retinal Pigmented Epithelial Cells) were grown in D-MEM/high glucose medium (Thermofisher) with 10% FBS, 1% Glutamine and 1% P/S.

Nutrient deprivation was achieved by growing cells in DMEM/F-12 or D-MEM/high glucose 1% P/S. Cells were cultured at 37 °C in a 5% CO_2_ atmosphere. Throughout the paper when we refer to starved cells, we mean 24 h starved cells.

### Constructs and cell transfection

The h*VPS39* coding sequence was cloned in the p3XFlag-CMV-14 plasmid. For Medaka experiments, h*VPS39* was cloned in the pCS2+ vector, as described in (70). HK2 cells were transfected using TransIT®-LT1 transfection reagent from Mirus, following manufacturing instructions. Cells were plated, transfected and reached 100% confluency after 24 h. Cells were then brought to quiescence by serum deprivation for 24 h, and thus collected after 48 h (24 h + 24 h) after transfection.

### Drug treatments

To induce ciliogenesis, HK2 cells were grown to 100% density and brought to quiescence by adding FBS-deprived medium for 24 h. To modulate autophagy cells were treated with drugs for 16 h in FBS-deprived medium (for a total time of 24 h of serum starvation). Drug concentrations: 50 nM Bafilomycin A1 (B1793, Sigma-Aldrich), 4 μM Tat-Beclin1 D11 (NBP2–49888 NovusBio) and 100 nM Rapamycin (R0395 from SIGMA).

### RNA interference

HK2 cells were transfected with the ON-TARGET plus smart pool siRNAs against the h*VPS39* transcript (L-014052-01 Dharmacon) or ON-TARGETplusNon-Targeting Pool (D-001810-10-20; Thermo Scientific Dharmacon), using INTERFERIN from Polyplus following manufacturing instructions. Cells were incubated in complete medium for 72 h and grown to 100% density, then they were starved with FBS-deprived medium for 24 h. Cells were then collected 96 h after transfection for western blot (WB) analyses, or they were fixed for IF staining.

### RNA extraction and real-time PCR

Total RNA from whole HK2 lysates was purified on RNeasy Columns (Qiagen). Total RNA from whole medaka embryos was purified using QIAzol Lysis Reagent (79 306, QIAGEN) and the miRNeasy Mini Kit (217 004, QIAGEN). cDNAs were obtained using the SuperScript First-Strand kit (Invitrogen). Real-time PCR methods were previously described (71). Primers used to amplify the h*VPS39* transcript were: Fw ACCACAGCAAACTCCACACC and Rv AAATCCGCTCTTCCTGGACC. The Hypoxanthine-guanine phosphoribosyltransferase (*HPRT*) transcript was used as reference for experiments in both human and medaka systems. Primers for h*HPRT* and Medaka *olHprt* were described in (71). For each experiment, three technical replicates were performed. Analyses were performed at least three times.

### WB analysis

HK2 cells were collected in a lysis buffer, composed by 20 mM TrisHCl at pH 8, 5% v/v glycerol, 138 mM NaCl, 2.7 mM KCl, 1% NP40, supplemented with proteases inhibitor (P8340, Sigma-Aldrich). The antibodies used for the analysis were: LC3B (NB100-2220, Novus Biologicals 1:1000), Phospho-S6 (2211, Cell Signaling 1:10 000), β-Actin (A5441, Sigma-Aldrich 1:1000), GAPDH (sc-515 381, Santa Cruz Biotechnology 1:2000), FLAG (F1804, Sigma-Aldrich 1:1000), SQSTM1 (H00008878-M01, Abnova 1:500), VPS39 (S-14) (sc-104 761, Santa Cruz Biotechnology 1:200). Secondary antibodies were from GE Healthcare Life Sciences. Analyses were performed at least three times.

### Immunofluorescence

Cells were fixed with cold methanol for 5 min. *VPS39*-silenced cells were fixed 96 h after the silencing while VPS39-overexpressing cells were fixed 72 h after transfection, blocking and permeabilization were achieved in 0.2% Triton X-100, 10% FBS in PBS. For LC3 detection blocking/permeabilization solution was performed with 0.5% bovine serum albumin (BSA), 0.05% saponin, 50 nM NH4Cl, 0.02% NaN3, in PBS 1X, pH 7.2–7.4). Primary antibodies were: ARL13b (17711-1-AP, Proteintech 1:1000), γ-tubulin (GTU-88, Sigma-Aldrich 1:5000), FLAG-M2 (F3165, Sigma-Aldrich 1:250), IFT20 (13615-1-AP, Proteintech 1:100), OFD1 (HPA031103, Sigma-Aldrich 1:800), LC3B (NB100-2220, Novus Biologicals 1:400). Secondary antibodies Alexa Fluor® IgG were from Thermo Fisher Scientific (1:800). Hoechst 33342 (14 522, Sigma-Aldrich 1:1000) was used to stain nuclei. Confocal fluorescence microscopy and image processing were performed, as described in (72). The significance of the results was calculated by Student’s t-test or Anova and reported as *P*-value.

Definition of the pericentriolar region. We acquired images by confocal microscopy and drew a cube of 2 μm^3^ around the centrosome to define the pericentriolar region, as described in (73). The acquisition of z-stack imagines was performed collecting 6 slices for sample.

### Medaka fish stocks

Medaka fish (*O. latipes*) of the cab strain were maintained in the in-house TIGEM facility (28 °C on a 14/10 h light/dark cycle). Embryos were staged, as described in (52).

Ethical statement: All studies on fish and mice were conducted in accordance with ARRIVE guidelines for animal research and approved by the Italian Ministry of Health; Department of Public Health, Animal Health, Nutrition and Food Safety in accordance to the law on animal experimentation (article 7; D.L. 116/92; protocol number: 389/2015-PR and 575/2017-PR). All animal treatments were reviewed and approved in advance by the Institutional Ethics Committee at the Telethon Institute of Genetics and Medicine (Pozzuoli, Italy).

### mRNAs injections

h*VPS39* mRNA was transcribed *in vitro* from pCS2+ plasmids using the mMESSAGE mMACHINE® High Yield Capped RNA Transcription Kit (SP6 Kit, 1340, Ambion) according to the manufacturer’s instructions. Three hundred nanograms of h*VPS39* mRNA was injected into medaka fertilized embryos at the one/two-cell stage, as described in (70).

### Drug treatments in medaka fish

Stage 32 embryos were dechorionated and incubated for 24 h in 100 μM CQ (Sigma-Aldrich), as described in (74). Drugs were diluted in the embryo medium. Control embryos were grown in the embryo medium. At least 3 independent experiments were performed for each condition.

### Immunofluorescence on medaka fish

The animals were subjected to anesthesia at st36 and then fixed with 4% Paraformaldehyde (PFA) in PTW (PBS and 0.1% TWEEN®20). The samples were included in 15% sucrose and 7.5% gelatin and rapidly frozen with N-Pentane and liquid nitrogen. Sections were incubated with 10% FBS, 2% BSA, 0.2% Triton X-100 in PBS and IF were performed, as described in (74). Primary antibody: acetylated-tubulin (T6793, Sigma-Aldrich 1:100). Secondary antibodies were Alexa Fluor® IgG (Thermo Fisher Scientific 1:500). Hoechst 33342 (14 522, Sigma-Aldrich 1:1000) was used to stain nuclei.

### Statistical analysis

The number of experimental replicates is indicated in each figure legend. In all experiments, significance of differences between groups was evaluated by one-way or two-way ANOVA with *post hoc* analysis, one-tailed or two-tailed Student’s t-test, proportion-test with *post hoc* analysis. *P* < 0.05 was considered significant. Quantitative data are presented as the mean ± SEM (standard error of the mean).

## Supplementary Material

Supplementary_Figures_ddaa029
