# Supplementary material for: The HOPS complex subunit VPS39 controls ciliogenesis through autophagy
Source: Hum Mol Genet. 2020 Feb 20;29(6):1018–29. doi: 10.1093/hmg/ddaa029 (PMC7158379; doi:10.1093/hmg/ddaa029)
Supplement: Supplementary_Figures_ddaa029 [file supplementary_figures_ddaa029.docx]

**SUPPLEMENTARY FIGURES**

**
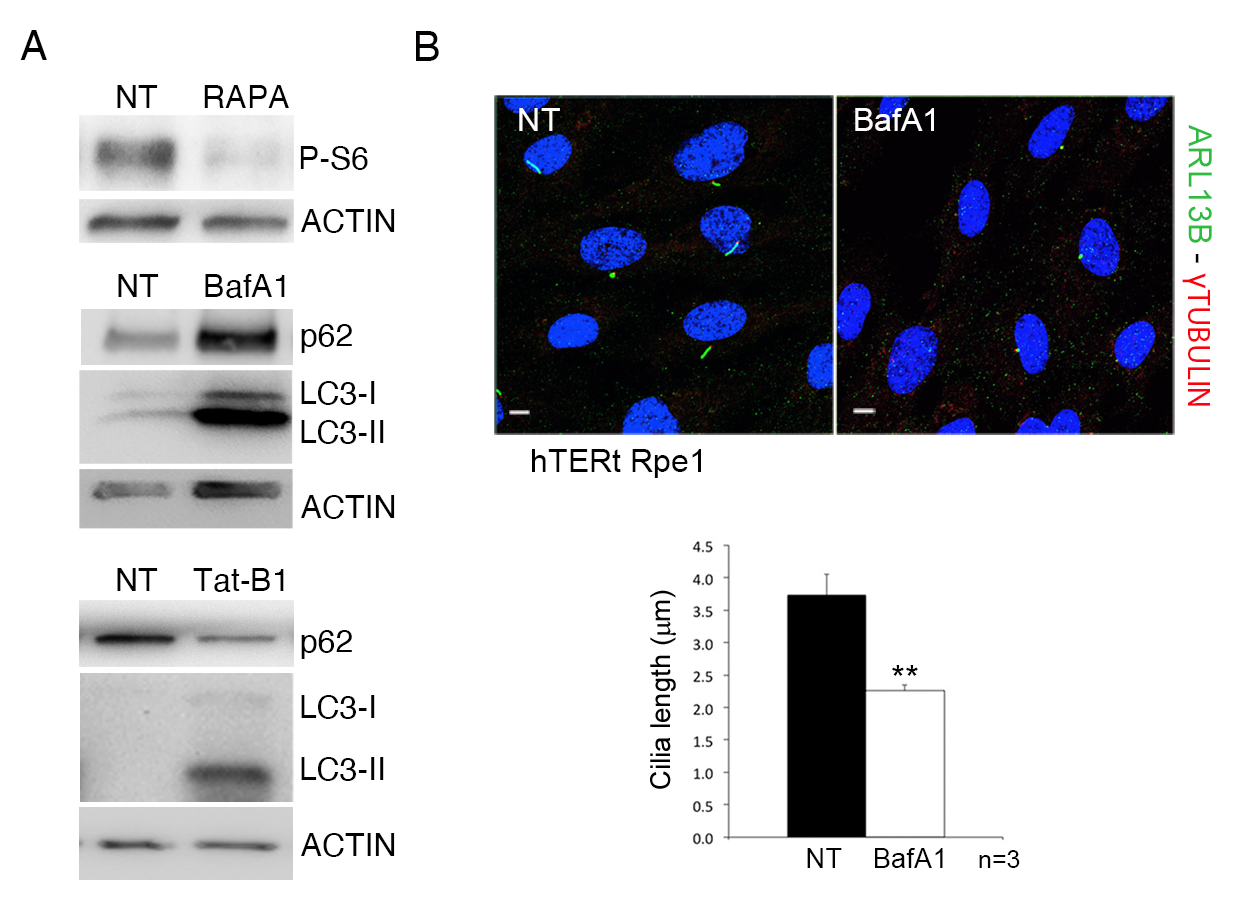
**

**Figure S1.** (A) Efficiency of autophagy modulators was confirmed on HK2 cells with different markers. To evaluate Rapamycin (RAPA) treatment (top) we used Phospho-S6 protein (P-S6) as marker of the mTORC1 pathway. BafilomycinA1 (BafA1) and Tat-Beclin1 (Tat-B1) treatments efficacy was tested using two markers of the autophagic flux, LC3 and p62 (middle and bottom panels, respectively). Each blot is representative of n=3 independent experiments. (B) The effect of BafA1 on cilia in hTERt Rpe1 cells was observed by IF with an antibody against ARL13b that decorates primary cilia. Cilia length was quantified and reported in the graph. Treated cells showed shorter cilia (white bar) compared to untreated (NT) controls (black bar). Bars= 10μm. Hoechst was used to stain nuclei. Data are presented as the mean + SEM. T-test was used to evaluate significance **pvalue <0,01. n=number of replicates.


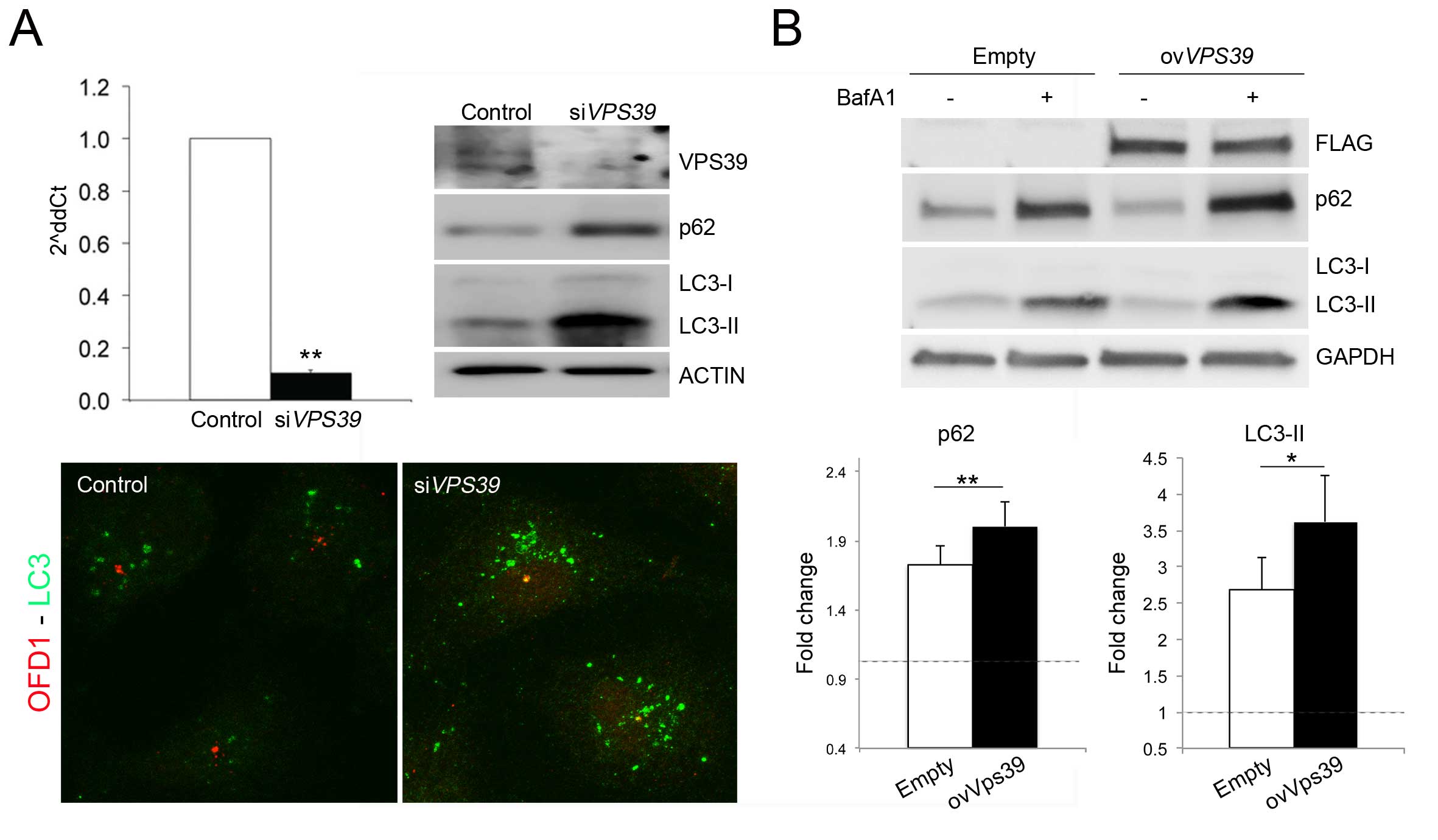


**Figure S2. VPS39 controls autophagy in HK2 cells.** (A) Upper panel. *VPS39* silencing was confirmed by Real-Time PCR and resulted on average in 90% reduction of the transcript level in si*VPS39-*treated cells (black bar) compared to controls (Contr, white bar). WB analysis showed that *VPS39*-silenced cells display higher levels of p62 and LC3-II compared to controls. Lower panel. IF analysis of LC3 puncta (green) showed accumulation of LC3 in *VPS39*-silenced cells (right) compared to control cells (left). OFD1 (red) was used to mark centrosomes. (B) Upper panel. WB analysis of LC3 and p62, markers of the autophagic pathway, in HK2 cells transiently transfected with the empty vector (Empty) or the 3xFLAG-VPS39 construct (ovVPS39) for 48h and treated (+) or not treated (-) with BafA1. Lower panel. Histograms show quantification of LC3-II and p62 protein levels under BafA1 treatment, values are expressed as the fold change compared with not treated samples (represented by the dashed line, value=1). n=3 independent experiments. Data are presented as the mean + SEM. *pvalue <0,05 and **pvalue <0,01.


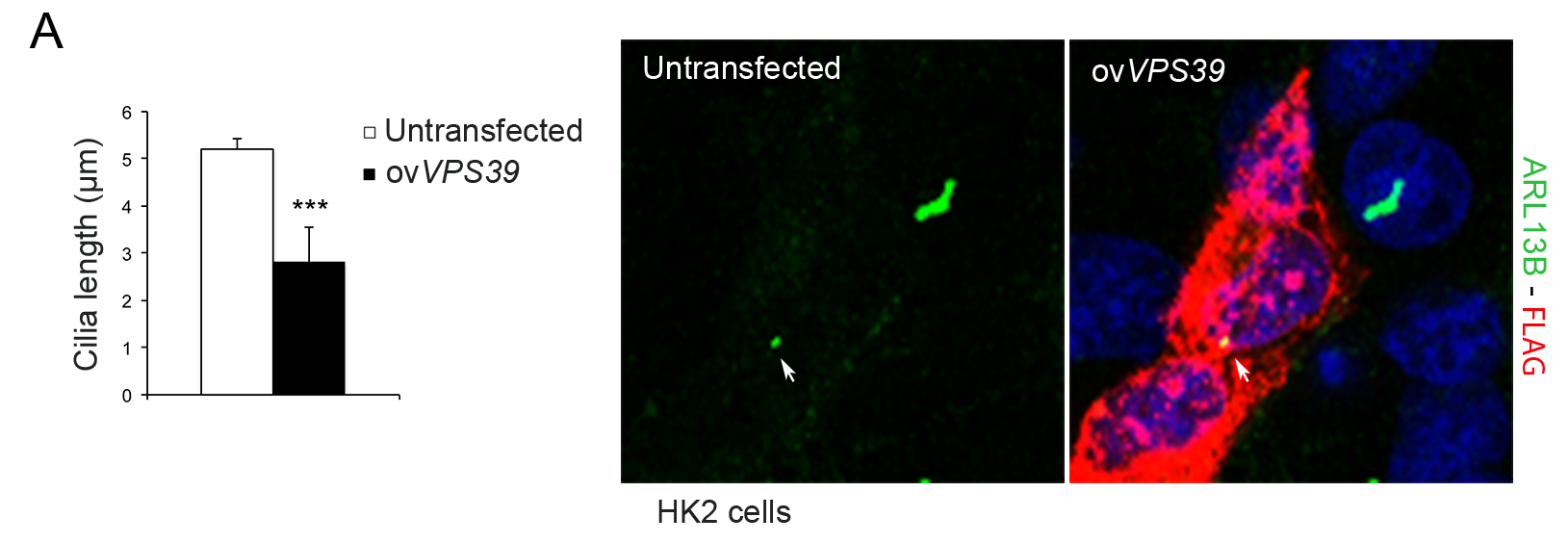


**Figure S3. VPS39 controls cilia elongation in HK2 cells.** Right. Representative confocal images of HK2 cells overexpressing 3XFlag-hVPS39 (red) stained with the anti-Arl13b antibody (green). Nuclei were stained with Hoechst dye (blue). Left. Histograms show quantification of cilia length. 3XFlag-hVPS39 overexpression (ovVps39 – black bar) resulted in shorter cilia compared to untransfected cells (Untransfected – white bar). Data are presented as the mean + SEM (n=3 independent experiments). T-test was used to evaluate significance ***pvalue <0,001.
